# Resuscitation with an Intact Cord Enhances Pulmonary Vasodilation and Ventilation with Reduction in Systemic Oxygen Exposure and Oxygen Load in an Asphyxiated Preterm Ovine Model

**DOI:** 10.3390/children8040307

**Published:** 2021-04-17

**Authors:** Praveen Chandrasekharan, Sylvia Gugino, Justin Helman, Carmon Koenigsknecht, Lori Nielsen, Nicole Bradley, Jayasree Nair, Vikash Agrawal, Mausma Bawa, Andreina Mari, Munmun Rawat, Satyan Lakshminrusimha

**Affiliations:** 1 Department of Pediatrics, Jacobs School of Medicine & Biomedical Sciences, University at Buffalo, Buffalo, NY 14260, USA; sfgugino@buffalo.edu (S.G.); jhelman@buffalo.edu (J.H.); carmonko@buffalo.edu (C.K.); lnielsen@buffalo.edu (L.N.); nkbradle@buffalo.edu (N.B.); jnair2@buffalo.edu (J.N.); mausmaba@buffalo.edu (M.B.); amaritor@buffalo.edu (A.M.); mrawat@upa.chob.edu (M.R.); 2 Department of Pediatrics, Loma Linda University, Loma Linda, CA 92350, USA; dragrawalmd@gmail.com; 3 Department of Pediatrics, Division of Neonatology, University of California Davis School of Medicine, Sacramento, CA 95817, USA; slakshmi@ucdavis.edu

**Keywords:** delayed cord clamping, oxygen exposure, preterm neonates

## Abstract

(1) Background: Optimal initial oxygen (O_2_) concentration in preterm neonates is controversial. Our objectives were to compare the effect of delayed cord clamping with ventilation (DCCV) to early cord clamping followed by ventilation (ECCV) on O_2_ exposure, gas exchange, and hemodynamics in an asphyxiated preterm ovine model. (2) Methods: Asphyxiated preterm lambs (127–128 d) with heart rate <90 bpm were randomly assigned to DCCV or ECCV. In DCCV, positive pressure ventilation (PPV) was initiated with 30–60% O_2_ and titrated based on preductal saturations (SpO_2_) with an intact cord for 5 min, followed by clamping. In ECCV, the cord was clamped, and PPV was initiated. (3) Results: Fifteen asphyxiated preterm lambs were randomized to DCCV (N = 7) or ECCV (N = 8). The inspired O_2_ (40 ± 20% vs. 60 ± 20%, *p* < 0.05) and oxygen load (520 (IQR 414–530) vs. 775 (IQR 623–868), p-0.03) in the DCCV group were significantly lower than ECCV. Arterial oxygenation and carbon dioxide (PaCO_2_) levels were significantly lower and peak pulmonary blood flow was higher with DCCV. (4) Conclusion: In asphyxiated preterm lambs, resuscitation with an intact cord decreased O_2_ exposure load improved ventilation with an increase in peak pulmonary blood flow in the first 5 min.

## 1. Introduction

In the delivery room, a depressed preterm neonate often needs positive pressure ventilation (PPV) with supplemental oxygen [1]. In a surfactant deficient state complicated by birth asphyxia, the current recommendations to start ventilation with 21–30% oxygen (O_2_) may not achieve target saturations (SpO_2_) [2,3,4]. Inability to achieve target SpO_2_ by 5 min is associated with poor outcomes in preterm infants. Birth asphyxia could increase pulmonary vascular resistance (PVR) and impair pulmonary vascular transition at birth. In a subgroup analysis of the Targeted Oxygen in the Resuscitation of Preterm Infants and their Developmental Outcomes (To2rpido) trial by Oei et al. <28-week gestation preterm infants resuscitated with 21% O_2_ had higher mortality due to respiratory failure, compared to 100% O_2_ [5]. Previously, we have shown that ventilation with 100% O_2_ (but not 21%), led to a decrease in PVR and increased systemic to pulmonary pressure gradient in preterm lambs [6]. Such impaired pulmonary vascular transition at birth could explain the higher mortality demonstrated by Oei et al. in <28-week infants resuscitated with 21% oxygen [5]. Although 100% O_2_ exposure in preterm lambs was associated with improved pulmonary blood flow and preductal SpO_2_, it also led to supraphysiological arterial oxygenation (PaO_2_) that could lead to hyperoxic injury [6]. With concerns of hyperoxia, higher oxidative injury, the use of 100% O_2_ is not recommended [2,3].

The feasibility of delaying the clamping of the cord and its benefits have been studied extensively [1,7,8,9,10,11,12,13,14,15]. However, optimal oxygenation during delayed cord claming (DCC) and ventilation is not known. Ventilation with an intact cord has been shown to improve cardiovascular transition in both translational animal models and human newborns [10,11,12,13,15,16,17,18,19,20,21,22,23,24]. It has been recently reported that the preductal SpO_2_ in neonates who had an intact cord was higher compared to the recommended SpO_2_ ranges obtained following early cord clamping (ECC) [13,16,22].

Currently, there are no concrete recommendations to delay the clamping of the cord and provide positive pressure ventilation in a depressed preterm neonate [2,3]. We conducted this pilot study to understand the effect of ventilation and supplemental oxygen with an intact cord using an asphyxiated preterm ovine model to address this knowledge gap. We aimed to study the effect of supplementing oxygen with and without an intact cord in the setting of perinatal acidosis, on preductal SpO_2_, oxygenation, ventilation, and hemodynamic parameters in the first 5 min of resuscitation.

## 2. Materials and Methods

The study was approved by the Institutional Animal Care and Use Committee, University of Buffalo, Buffalo New York, USA and followed the ARRIVE guidelines. Time-dated ewes (127–128 d, gestation), approximately equivalent in lung maturity to preterm neonate ≈28-week gestation were used in this study. The ewes did not receive antenatal steroids. After an overnight fast, the ewes were anesthetized, a caesarean section was performed, and fetal lambs were partially exteriorized. These fetal lambs were instrumented while in placental circulation as described previously [6]. Instrumentation included placement of the right jugular and carotid lines for access, pressure monitoring, and blood draws. A left carotid artery probe was placed to monitor blood flows. The left pulmonary artery probe and ductal arteriosus probe were placed to monitor blood flows. The lambs were intubated, and the lung fluid was drained by gravity.

Asphyxia was induced by umbilical cord occlusion until the heart rate (HR) reached <90 bpm. Once the target HR was achieved, the fetus was randomized to early cord clamping and ventilation (ECCV) or delayed cord clamping and ventilation (DCCV) for 5 min. Our primary objective was to achieve a composite outcome of HR ≥100 bpm and SpO_2_ ≥80% by 5 min. For this reason, in DCCV, the cord was clamped only after 5 min unless the fetus had a drop in HR <60 bpm and ventilation continued. To simulate a real-life clinical scenario, the initial supplemental oxygen (O_2_) concentration was between 30–60% in both groups and not changed during the first 2 min of PPV. After 2 min, the O_2_ concentration was adjusted based on the neonatal resuscitation program (NRP) recommended target preductal SpO_2_. The titration of O_2_ was proportional to the difference between observed SpO_2_ and target SpO_2_ and was performed every min. In order to obtain an accurate and continuous recording of preductal SpO_2_, two preductal probes (on the tongue and the right upper limb) were placed.

Oxygen load (OL) was calculated using the following formula, OL = (VT × FiO_2_)/kg, where VT is tidal volume and FiO_2_ is a fraction of inspired oxygen. Total OL was calculated using the summation of breaths for 5 min as defined previously [25].

In a depressed, asphyxiated and surfactant-deficient preterm lamb with HR < 90 bpm, our primary objective was to evaluate if titrating supplemental O_2_ with (DCCV) and without an intact cord (ECCV) would lead to a higher rate of success in achieving target saturations as recommended by neonatal resuscitation program (NRP). The secondary objectives were to assess gas exchange and systemic and pulmonary hemodynamics along with oxygen load in the first 5 min of resuscitation.

Parametric data are presented as mean and standard deviation and analyzed by ANOVA. Non-parametric data are presented as median and interquartile range and analyzed by the Kruskal-Wallis test. The significance was set at a probability of less than five percent.

## 3. Results

Fifteen preterm lambs were randomized to DCCV (N-7) and ECCV (N-8). The characteristics are shown in Table 1 and were similar.

The asphyxiated preterm model had perinatal acidosis as shown in Table 1, which improved significantly with DCCV (7.02 ± 0.08) compared to ECCV (6.96 ± 0.13). The difference in pH was statistically significant throughout the first 5 min of PPV (*p* = 0.02). The HR improved in both groups by 5 min—DCCV (180 ± 20 bpm) and ECCV (188 ± 30 bpm). The mean blood pressures in both groups by 5 min were similar (DCCV (40 ± 10 mmHg) and ECCV (44 ± 15 mmHg)).

### 3.1. Oxygenation

**Figure 1:**
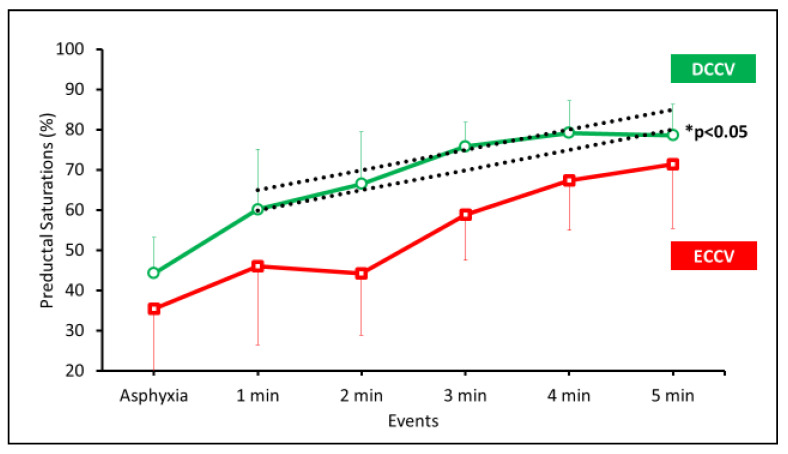
Preductal SpO_2_ are shown on the y-axis and the events or duration of positive pressure ventilation (PPV) on the x-axis. The data are represented as average and standard deviation. The SpO_2_ between ECCV (early cord clamping followed by ventilation) and DCCV (delayed cord clamping with ventilation) was significantly different (* *p* < 0.05 by ANOVA).

A higher percentage of lambs achieved preductal saturation targets in the DCCV group (75%) in the first 5 min compared to ECCV (60%). However, this result was not statistically significant (p-0.065). Preductal SpO_2_ was significantly higher in the DCCV group compared to ECCV (p-0.01) (Figure 1).

**Figure 2:**
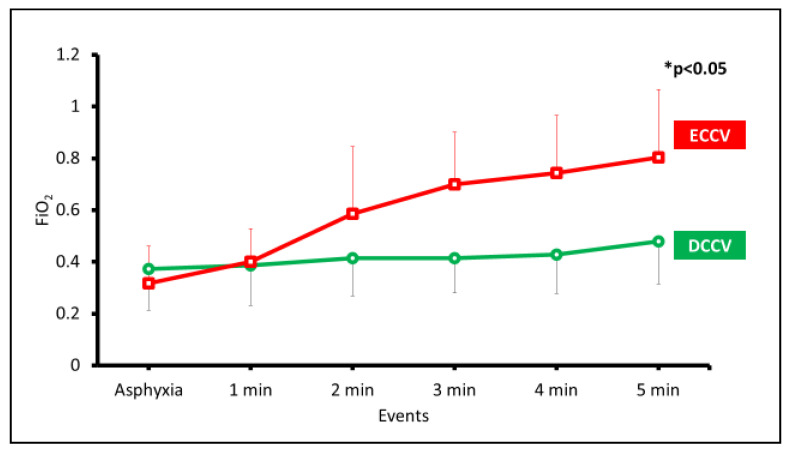
Fraction of inspired oxygen (FiO_2_) is shown on the y-axis and the events or duration of PPV on the x-axis. The data are represented as average and standard deviation. The difference between ECCV and DCCV was significantly different (* *p* < 0.05 by ANOVA).

The required oxygen concentration to achieve these target SpO_2_ were significantly higher in the ECCV group compared to the DCCV group (Figure 2).

**Figure 3:**
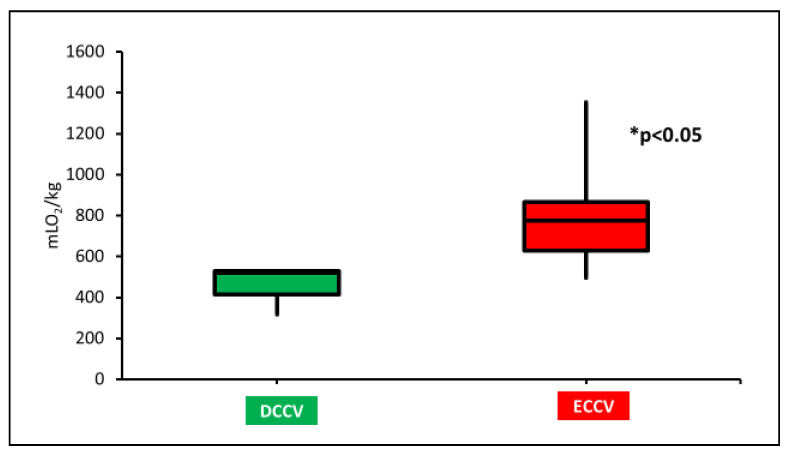
The oxygen load (OL) in milliliters of oxygen per kilogram is shown on the y-axis. The box and whiskers plot shows a significantly higher OL with ECCV compared to DCCV. OL in ECCV was significantly higher compared to DCCV (* *p* < 0.05 by Kruskal-Wallis test).

Oxygen load (OL)—as mentioned previously, OL was calculated using the tidal volume (TV) per kg in the first 5 min. The average TV in ECCV and DCCV were 6.0 ± 1.5 mL/kg and 6.0 ± 2.3 mL/kg, respectively. The OL with DCCV (520 (414–530) mLO_2_/kg) was significantly lower compared to ECCV (775 (630–867) mLO_2_/kg) (*p* = 0.03) as shown in Figure 3.

**Figure 4:**
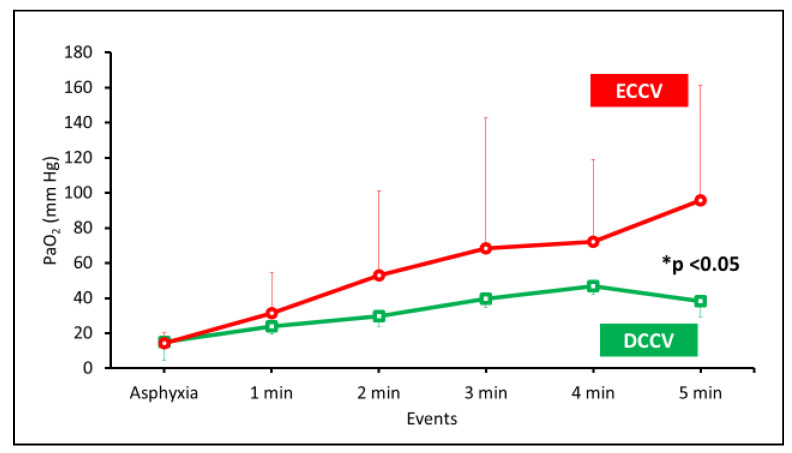
The PaO_2_ in mm Hg is shown on the y-axis and the events or duration of PPV on the x-axis. The PaO_2_ was significantly lower in DCCV (* *p* < 0.05 by ANOVA).

Arterial oxygenation (PaO_2_)—despite the lower percentage of SpO_2_ targets with ECCV, the PaO_2_ was significantly higher (95 ± 65 mmHg) compared to DCCV (38 ± 9 mmHg, p-0.007) (Figure 4). The variability in arterial oxygenation with DCCV was minimal compared to ECCV, as shown by the standard deviations in Figure 4. The PaO_2_/FiO_2_ ratio at 5 min of PPV was 98 ± 50 in the DCCV group and 135 ± 87 mmHg in the ECCV group (p-0.4).

### 3.2. Ventilation

**Figure 5:**
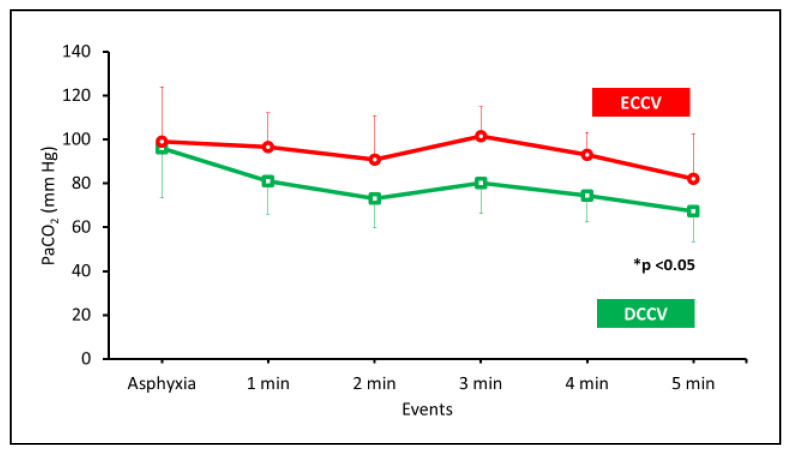
The arterial carbon dioxide (PaCO_2_ in mmHg) is shown on the y-axis and the events on the x-axis. The PaCO_2_ was significantly lower in DCCV compared to ECCV. (* *p* < 0.05 by ANOVA).

As mentioned previously, despite similar TV and ventilation rates in both groups in the first 5 min, the arterial carbon dioxide (PaCO_2_) levels were significantly lower with DCCV compared to ECCV (Figure 5).

### 3.3. Hemodynamics

**Figure 6:**
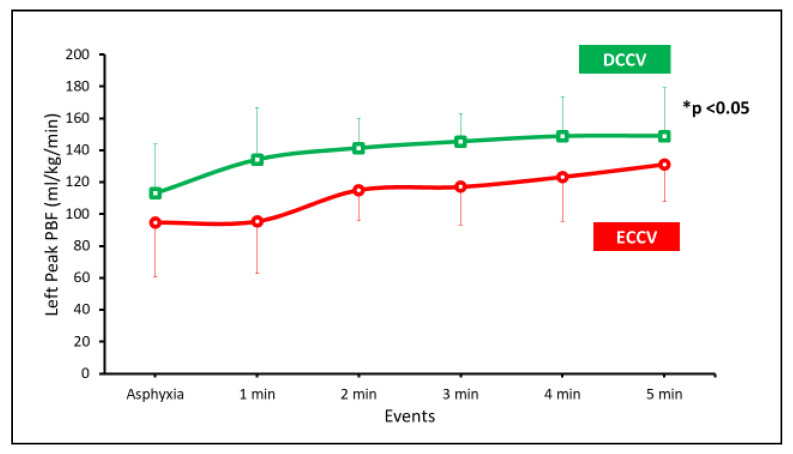
The left peak PBF is shown on the y-axis and the events on the x-axis. The PBF was significantly higher with DCCV compared to ECCV (* *p* < 0.05 by ANOVA).

Pulmonary blood flow—the peak left pulmonary blood flows (PBF) were significantly higher in the DCCV group compared to ECCV (Figure 6).

**Figure 7:**
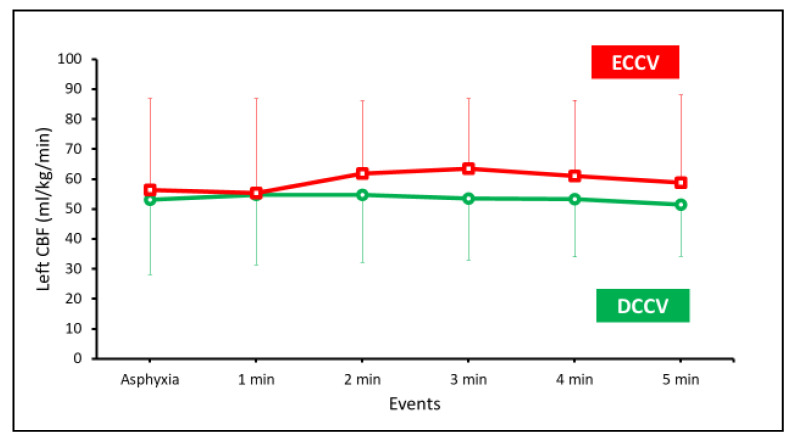
The left peak CBF is shown on the y-axis and the events or duration of PPV on the x-axis.

Carotid blood flow—the peak left carotid blood flow (CBF) was not different between the two groups as shown in Figure 7.

## 4. Discussion

**Figure 8:**
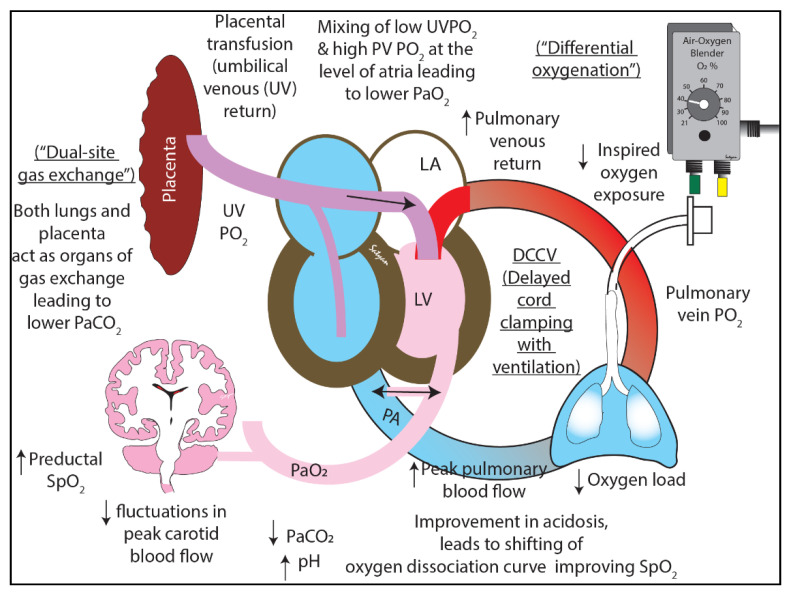
The benefits of delayed cord clamping and ventilation (DCCV) for 5 min in a lamb model of preterm asphyxia. With positive pressure ventilation and an intact cord, there was a mixing of lower umbilical vein oxygen tension (UVPO_2_) with higher pulmonary venous oxygen tension (PVO_2_) leading to overall systemic lower oxygen tension (PaO_2_)—“differential oxygenation” with alveolar hyperoxia but systemic normoxia. With the placenta and lungs simultaneously acting as organs of gas exchange, the arterial carbon dioxide (PaCO_2_) was lower (“dual-site gas exchange”). Copyright Satyan Lakshminrusimha.

In a depressed preterm neonate with HRs between 60–90 bpm, lower supplemental O_2,_ as currently recommended by NRP, may not achieve the pre-specified target SpO_2_ or sustain pulmonary vasodilation. An alternative strategy is to ventilate with an intact cord and higher concentrations of supplemental O_2,_ especially in the setting of perinatal acidosis. Perinatal acidosis increases PVR. Low FiO_2_ may not increase alveolar oxygen adequately to induce pulmonary vasodilation in preterm infants with high PVR. A combination of increased FiO_2_ and resuscitation with an intact cord results in “transient alveolar hyperoxia” without systemic hyperoxia due to “buffering” of PaO_2_ by umbilical venous return (Figure 4 and Figure 8). Such “differential oxygenation” may be an effective concept for the resuscitation of depressed preterm infants that do not show spontaneous respiratory activity. The second advantage of resuscitation with an intact cord is “dual-site” gas exchange enabling CO_2_ elimination in both the lungs and placenta.

In our pilot study, we tested our concept of “differential oxygenation” and “dual-site gas exchange” in a surfactant-deficient asphyxiated preterm lamb. We assessed the success of achieving target preductal saturation, gas exchange, and hemodynamics during ventilation with an intact cord as compared to the current standard of early cord clamping followed by resuscitation.

Arterial PaCO_2_ levels were significantly lower in the DCCV group despite similar inflation pressures, tidal volumes, and ventilator rates (Figure 5). We speculate the contribution of placental gas exchange during resuscitation with an intact cord for this decrease. More efficient ventilation leads to earlier normalization of pH with DCCV. Early normalization of PaCO_2_ increases pulmonary blood flow and avoids cerebral hyperemia.

The success in achieving the target preductal SpO_2_ in asphyxiated preterm lambs was similar in DCCV (75%) compared to ECCV (60%) lambs, although DCCV lambs required significantly lower FiO_2_. Previous clinical studies have shown improved SpO_2_ in the first few minutes after birth with an intact cord compared to immediate cord clamping [16,18,22]. Recently, the preductal SpO_2_ curves in spontaneous breathing term infants with an intact cord for a min were developed by Padilla-Sánchez et al. These curves show increased SpO_2_ compared to immediate cord clamping, as shown in a study published by Dawson et al. [13].

Our study’s striking feature was that the arterial oxygenation was much lower with decreased variability (as observed by lower standard deviations) in the DCCV group compared to the ECCV in the first 5 min. We speculate that the mixing of higher PO_2_ pulmonary venous return with lower PO_2_ in the umbilical venous return in the atrium, reduces systemic oxygenation as evidenced by lower PaO_2_/FiO_2_ ratios in the DCCV lambs. We speculate that, with a mix of PO_2_ from the placenta and lungs, these term babies would have had lower PaO_2_ compared to infants breathing with their umbilical cord cut despite higher SpO_2_.

Despite lower PaO_2_ in DCCV, the SpO_2_ was significantly higher compared to ECCV. We speculate that the better SpO_2_ reading in the DCCV lambs is due to two factors: better perfusion and prevention of a rightward shift in hemoglobin oxygen dissociation curve due to the rapid correction of acidosis. Asphyxia with combined respiratory and metabolic acidosis shifts the hemoglobin-oxygen dissociation curve leading to lower SpO_2_ for a given PaO_2_. We speculate that with better ventilation (lower PaCO_2_) and higher pH in the DCCV group, SpO_2_ values were higher despite lower PaO_2_ levels in DCCV compared to ECCV [26]. By 5 min, the highest PaO_2_ in DCCV was 38 ± 9 mmHg compared to ECCV, where it was 96 ± 65 mmHg. In a study, involving term neonates, Kapadia et al. have shown that perinatal acidemia and post-resuscitation hyperoxemia (defined as an initial PaO_2_ of >100 mmHg) were associated with a higher incidence of hypoxic ischemic encephalopathy [27]. It is prudent to avoid high systemic PaO_2_ and PaCO_2_ values in asphyxiated preterm infants to limit cerebral hyperemia and oxidative reperfusion injury. Such injury can also predispose infants to intraventricular hemorrhage (IVH) in preterm neonates [28,29].

The oxygen exposure in the DCCV group was also much lower compared to the ECCV group. Hyperoxia in the delivery room is associated with an inflammatory response in the brain and the heart [30,31,32,33,34]. In a study involving an ovine model, hyperoxic resuscitation after fetal asphyxia led to the cerebral inflammatory response [34]. Secondary to the immature antioxidant response, oxidative injury in preterm infants can modify DNA methylation patterns, thus affecting gene expressions [35]. Lorente-Pozo et al. studied the effect of OL in preterm infants <32 weeks who were resuscitated with supplemental O_2_ on DNA methylome [25]. The study concluded that OL during resuscitation altered DNA methylome that could potentially alter genes related to cell cycle progression, oxidative stress, and DNA repair [25]. In this study, the median OL during stabilization was 644 mLO_2_/kg, and >500 mLO_2_/kg could alter the methylation pattern. In our study, the OL in the first 5 min of resuscitation was 520 mLO_2_/kg and was significantly lower than ECCV, which had an OL of 775 mLO_2_/kg. In this asphyxiated model, the decreased OL observed in the DCCV group can decrease oxidative injury.

The significant improvement in peak left pulmonary blood flow observed in the DCCV was similar to a previous study by Bhatt et al. in a preterm non-asphyxiated ovine model [19]. The increase in PBF could possibly be secondary to stable hemodynamics, better pH with lower PaCO_2_ in the DCCV group. Arterial oxygenation plays a great role in pulmonary vascular transition. Although the change point (PaO_2_ value) for pulmonary vascular transition in an asphyxiated preterm neonate/model remains unknown, we have previously shown that in a non-asphyxiated preterm model, a PaO_2_ of 31 ± 0.7 mmHg led to a drop in PVR [36]. In our study, we speculate that despite the lower PaO_2_ observed in the arterial blood, higher alveolar PAO_2_ helped overcome hypoxic pulmonary vasoconstriction and an improvement in pulmonary vascular transition in the DCCV group [26]. Custer et al. have shown that alveolar hypoxia could worsen hypoxic pulmonary vasoconstriction leading to a redistribution of pulmonary blood flow in a newborn ovine model [37]. Moreover, acidosis exacerbates hypoxic pulmonary vasoconstriction and better pH in DCCV could have ameliorated this effect, thus, leading to improved pulmonary vascular transition compared to ECCV.

Our study had some limitations. The data presented here are only for the first 5 min to compare the parameters while ventilating with and without an intact cord. We did not perform a sample size/power calculation for this pilot study. We did not collect markers of oxidative stress injury and used OL as a surrogate. The study was done in a controlled setting with multiple experienced personnel and may not be the case in real-life scenarios. Species differences do exist, but this asphyxiated preterm model with surfactant-deficient lungs has been widely used to study gas exchange and hemodynamics in the setting of placental transfusion. Since this was a pilot study and the recommended initial supplemental oxygen range by International Liaison Committee on Resuscitation is 30–60%, we chose random initial oxygen concentration with an average initial O_2_ concentration within the specified range. The distribution was similar in both ECCV and DCCV groups as shown in Figure 2.

## 5. Conclusions

In this pilot study, in an asphyxiated preterm ovine model with perinatal metabolic acidosis, ventilation with an intact cord for 5 min (DCCV), decreased oxygen exposure, decreased oxygen load, with improved ventilation, while increasing peak pulmonary blood flow compared to immediate clamping of the cord and ventilation (ECCV). In the future, we intend to study the effect of DCCV with low (30–60%) and high (60–100%) oxygen exposure and its effect on oxidative injury and its impact on pulmonary and systemic hemodynamics. Clinical trials evaluating the resuscitation of extremely preterm infants with an intact cord at different concentrations of supplemental oxygen are warranted.

## Figures and Tables

**Table 1:** Characteristics of preterm lambs.

Characteristics	ECC + V (N = 8)	DCC + V (N = 7)
Gestational age (days)	127 ± 0.52	128 ± 0.84
Female (N)	4	3
Birth weight (kg)	3.3 ± 0.63	3.3 ± 0.70
Born by multiplicity (N)	Twin–6	Twin–4
Heart rate at asphyxia (bpm)	88 ± 8	86 ± 10
Mean blood pressure at asphyxia (mmHg)	36 ± 8	34 ± 10
pH before resuscitation	7.04 ± 0.08	7.0 ± 0.08
PaCO_2_ before resuscitation( mmHg)	90 ± 25	101 ± 23
PaO_2_ before resuscitation (mmHg)	14 ± 6	15 ± 11

Data presented as numbers or as average and standard deviation. ECC—early cord clamping, DCC—delayed cord clamping, V—ventilation. PaCO_2_—arterial carbon dioxide, PaO_2_—arterial oxygenation.
